# Inhibition of NKCC1 Attenuated Hippocampal LTP Formation and Inhibitory Avoidance in Rat

**DOI:** 10.1371/journal.pone.0106692

**Published:** 2014-11-04

**Authors:** Meng Chang Ko, Min Chong Lee, Tamara G. Amstislavskaya, Maria A. Tikhonova, Yi-Ling Yang, Kwok-Tung Lu

**Affiliations:** 1 Department of Life Science, National Taiwan Normal University, Taipei, Taiwan; 2 Laboratory of Biological Psychiatry, State Research Institute of Physiology and Fundamental Medicine SB RAMS, Novosibirsk, Russia; 3 Department of Biochemical Science and Technology, National Chia-Yi University, Chia-Yi, Taiwan; Radboud University, Netherlands

## Abstract

The loop diuretic bumetanide (Bumex) is thought to have antiepileptic properties via modulate GABA_A_ mediated signaling through their antagonism of cation-chloride cotransporters. Given that loop diuretics may act as antiepileptic drugs that modulate GABAergic signaling, we sought to investigate whether they also affect hippocampal function. The current study was performed to evaluate the possible role of NKCC1 on the hippocampal function. Brain slice extracellular recording, inhibitory avoidance, and western blot were applied in this study. Results showed that hippocampal Long-term potentiation was attenuated by suprafusion of NKCC1 inhibitor bumetanide, in a dose dependent manner. Sequent experiment result showed that Intravenous injection of bumetanide (15.2 mg/kg) 30 min prior to the training session blocked inhibitory avoidance learning significantly. Subsequent control experiment's results excluded the possible non-specific effect of bumetanide on avoidance learning. We also found the phosphorylation of hippocampal MAPK was attenuated after bumetanide administration. These results suggested that hippocampal NKCC1 may via MAPK signaling cascade to possess its function.

## Introduction

The cation-chloride cotransporters (CCCs) are intrinsic membrane proteins that transport chloride ions, together with sodium and/or potassium ions across the plasma membrane [1]. The CCCs are divided into two main branches including the sodium-coupled CCC branch and the potassium-coupled CCC branch. The sodium-coupled CCC branch that comprises the Na-(K)-Cl-cotransporters NCC, NKCC1, and NKCC2 load chloride ions into the cell to raise [Cl^−^]_i_ above its electrochemical equilibrium. In adult neurons, the level of intracellular chloride is low and the reversal potential for chloride currents is near the neuron's resting membrane potential. Minor changes in the intracellular chloride concentration can significantly affect the strength, and even polarity, of GABAergic neurotransmission [2], [3]. In addition to setting the direction of GABA_A_ receptor-mediated currents, the intracellular chloride concentration is also an important osmotic determinant of cell volume [4]. Neurons, glia, and most other cells in the brain alter their cellular chloride concentration to defend their cell volume against fluctuations of extracellular osmolality and/or intracellular solute content-perturbations that can imperil their structural integrity [5]–[8].

Bumetanide is a loop diuretic which is commonly used for the treatment of edema associated with congestive heart failure, hepatic and renal disease for decades [9], [10]. Unlike another well-known loop diuretics furosemide, bumetanide has an approximately 500-fold greater affinity for NKCC1 (inhibition constant [Ki] of approximately 0.1 µM) than for KCC2 (Ki of approximately 25–50 µM). Furosemide inhibits NKCC1 and KCC2 with equal potency (Ki of approximately 25–50 µM). Therefore, at low doses (2–10 µM), bumetanide is a relatively specific inhibitor of NKCC1 in some specific cellular assay [11] which made it an ideal tool for studying the role of NKCC1 on hippocampus function. Recent studies also suggested some loop diuretics such as antiepileptic agent [1], [12], [13], [14]. Given that loop diuretics possibly act as antiepileptic agents that enhance GABA_A_ inhibition, we sought to investigate whether they also mediate the learning and memory function of hippocampus. Towards that end, brain slice extracellular recording, western blot and inhibitory avoidance paradigm were performed to evaluate the possible role of NKCC1 on hippocampal function.

## Materials and Methods

### Animals

Adult male Wistar rats (obtained from the animal center of National Taiwan University) weighing between 250 and 350 g were used. Animals were housed in group cages of four rats each in a temperature (24°C) - controlled animal colony, with continuous access to food and water. They were maintained on a 12∶12 light-dark cycle with lights on at 0700 hrs. All behavioral procedures took place during the animal light cycle. All procedures were conducted in accordance with the National institutes of Health Guide for Care and Use of Laboratory Animals and the guidelines set forth by the local institutional animal care and use committee (IACUC) at the National Taiwan Normal University. All efforts were made to minimize the animal numbers, which are required to produce meaningful experimental data.

### Drugs administration

For inhibitory avoidance, bumetanide was dissolved in 0.5% saline with 0.5N NaOH [15] and injected intravenously 30 min prior to the training session (15.2 mg/kg, equivalent to 400 µM). The chosen doses of bumetanide were based on our previous experiments [16], [17]. The test was carried out 24 hrs later. For extracellular recording, bumetanide was first dissolved in 100% DMSO to make a 10 mM stock solution and diluted to 5, 10, and 20 µM by ACSF [18].

### Inhibitory avoidance task

A conventional one-way inhibitory avoidance learning task was used to measure the retention performance in rats. It included the training phase and testing phase. Both of them were conducted between 9:00 a.m. and 4:00 p.m. Before experimentation, rats were kept in a dim room for 1 hr for acclimation. During the training phase, the rat was placed at the far end of the illuminated compartment facing away from the door. As the rat turned around, the door was opened. When the rat entered the dark compartment, the door was closed and a 1.2-mA/1-sec foot-shock was given. The animal was then removed from the dark compartment and returned to its home cage. The retention test was given 24 hr later. The animal was again placed into the illuminated compartment and the latency to step into the dark compartment was recorded as the measure of retention performance. The ceiling score was assigned as 600 sec.

### Locomotor activity monitoring

Rats were placed at the centre of a cubic chamber (48 cm x 48 cm x 48 cm). The animal's horizontal activity and stereotypic behaviors were monitored and recording by commercialized behavior monitoring system (Ethovision, USA). All animals were habituated to the test room for at least 20 min before the start of the session, and the test session was lasted for 5 min. The test room was dimly illuminated with indirect white light.

### Brain slice extracellular recording

In separate *in vitro* experiments, the rats were decapitated and the brains were quickly removed from the skull. Coronal slices normally cut into 400 µm thick by using vibrotome. The appropriate slices were placed in a beaker of artificial cerebrospinal fluid (ACSF). The ACSF was oxygenated continuously with 95% O_2_/5% CO_2_ to maintain the pH at 7.3–7.5. The ACSF consists of 117 mM NaCl, 4.7 mM KCl, 2.5 mM CaCl_2_, 1.2 mM MgCl_2_, 25 mM NaHCO_3_, 2 mM NaH_2_PO_4_ and 11 mM glucose. The slices were kept at room temperature for at least 1 hr for stabilization before recording. A single slice was then transferred to the recording chamber where it is held submerged between two nylon nets and maintained at 32 ± l°C. The chamber consists of a circular well a low volume (1–1.5 ml) and was perfused constantly at the rate of 3–4 ml/min. A bipolar stimulating electrode (SNE-2OOX, Kopf Instrument, Tujunga, CA, USA) was placed into the external capsule. Field excitatory postsynaptic potential (fEPSP) and population spike was recorded by using glass microelectrodes fined with 3M NaCl. Square-wave pulses of 200 ms were delivered at 20 s interval. The stimulation voltage was adjusted individually for each experiment to produce fEPSP, which are 30–40% of the maximal response that could be evoked. The strength of synaptic transmission was quantified by measuring the initial slope of the fEPSP. Before recording, input/output curves were calculated to set the optimal stimulation intensity which was adjusted to 50% of the evoked maximal response amplitude [19], [20].

### Western blot analysis

Animals were sacrificed by decapitation 1 hr after the training session. The hippocampus was collected and sonicated briefly in ice-cold buffer: 50 mM Tris–HCl (pH 7.8), 50 mM NaCl, 10 mM EGTA, 5 mM EDTA, 2 mM sodium pyrophosphate, 4 mM para-nitrophenylphosphate, 1 mM sodium orthovanadate, 1 mM PMSF, 20 ng/ml leupeptin, and 4 ng/ml aprotinin. Following sonication, the soluble extract was obtained after pelleting the crude membrane fraction in a centrifuge at 50,000 g at 4°C. Protein concentration in the soluble fraction was then measured using a Bradford assay with bovine serum albumin as the standard. Equivalent amounts of protein for each sample were resolved in 10% sodium dodecyl sulfate (SDS)–polyacrylamide gels, blotted electrophoretically to PVDF membranes and blocked overnight in 5% skim milk (Cell Signaling Technology, Inc., USA). Blots were incubated with antiphospho-MAPK antibody (New England Biolabs, USA), anti-MAPK antibody (BD Transduction Laboratories, USA), Band detection was performed with an enhanced chemiluminescence Western blotting analysis system (RPN 2108; Amersham International, Amersham, UK) [21].

### Statistics

Mann-Whitney U-tests were used for the analyzing of behavioral data. For the electrophysiological experiments, all data were expressed as mean ± S.E.M. For western blot experiment, statistical analysis was performed using one-way ANOVA followed with the Student's t-test for further comparison. Between-group comparisons were made using two-tailed *t* tests for independent samples. The criterion for significance for all comparisons was *p*<0.05.

## Results

### Experiment-1: Suprafusion of bumetanide suppressed *in vitro* hippocampal LTP formation in a dose dependent manner

**Figure 1:**
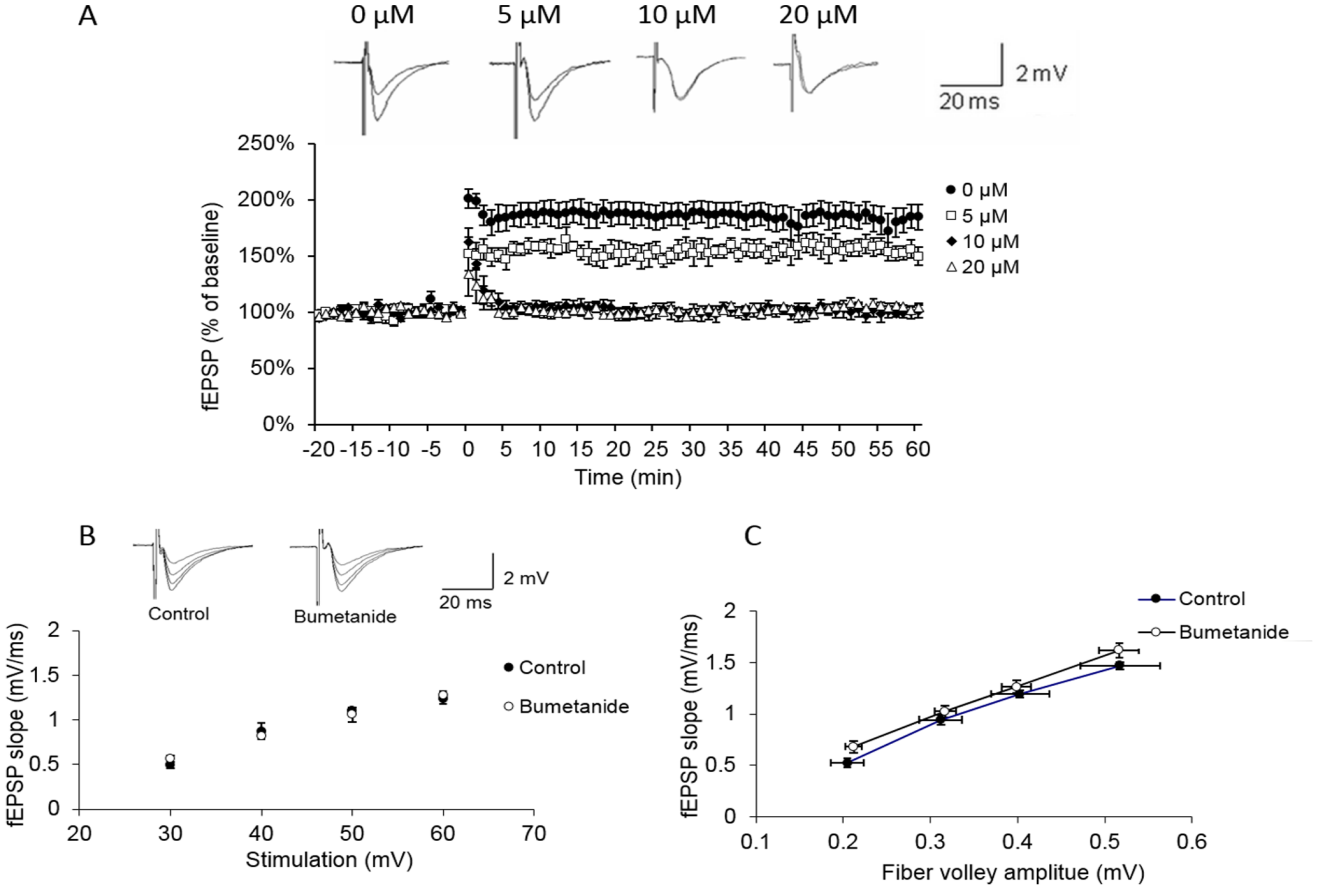
Superfusion of bumetanide blocked the formation of hippocampal LTP in a dose dependent manner. (A) From Left to right, representative superimposed traces of fEPSPs recorded extracellularlly under control conditions and after exposure to 0, 5, 10, and 20 µM bumetanide. Application of 10 µM bumetanide blocked LTP formation in hippocampus CA1 Schaffer collateral fiber. Bumetanide was applied from −10 min∼10 min. n = 10 for each trial (B) fEPSP recorded in the stratum radiatum and evoked by stimulation of the Schaffer collateral—commissural pathway with different intensities in vehicle-treated and bumetanide-treated slices (10 µM). fEPSP slopes are comparable between vehicle-treated (filled circle, n = 8) and bumetanide-treated (open circle, n = 8) for a given range of stimulus intensities. (C) Fiber volley amplitudes are similar between vehicle-treated and bumetanide-treated (10 µM) slices for a given range of stimulus intensities.

Hippocampal long-term potentiation (LTP) has been widely accepted as a candidate cellular mechanism for associative learning and memory. In order to investigate the possible role of NKCC1 on hippocampus function, brain slices containing hippocampus were subjected to extracellular recording. High frequency stimulus (100 Hz) was applied in a 20 second interval for a total of three times. Vehicle or bumetanide was applied 10 min prior to stimulation and sustained for 10 min after stimulation. Resulted showed that suprafusion of bumetanide blocked hippocampal LTP formation comparing with the vehicle group (vehicle group: 187% ± 11% bumetanide group: 102% ± 8%, p<0.001) (5 µM: 154% ± 8%, 20 µM: 105% ± 2%). Our results also showed that basal synaptic transmission is preserved in bumetanide treated rats. We first analyzed basal synaptic transmission by applying isolated stimuli of increasing intensity to the Schaffer collaterals (Figure 1B). Input/output curves for extracellular fEPSP were indistinguishable between slices from vehicle-treated group and bumetanide-treated. For a range of stimulation intensities, the slopes of bumetanide-treated slice's fEPSP responses were not significantly different from the fEPSP responses of vehicle-treated slices. Likewise, measurements of the fiber volleys amplitude from vehicle and bumetanide slices were similar (P>0.05), and there was no difference in an input/output curve (Fig. 1C). This result indicates that the given dose of bumetanide treatment does not modify the basal synaptic transmission at the postsynaptic level.

### Experiment 2: Systemic administration of bumetanide blocked inhibitory avoidance learning

**Figure 2:**
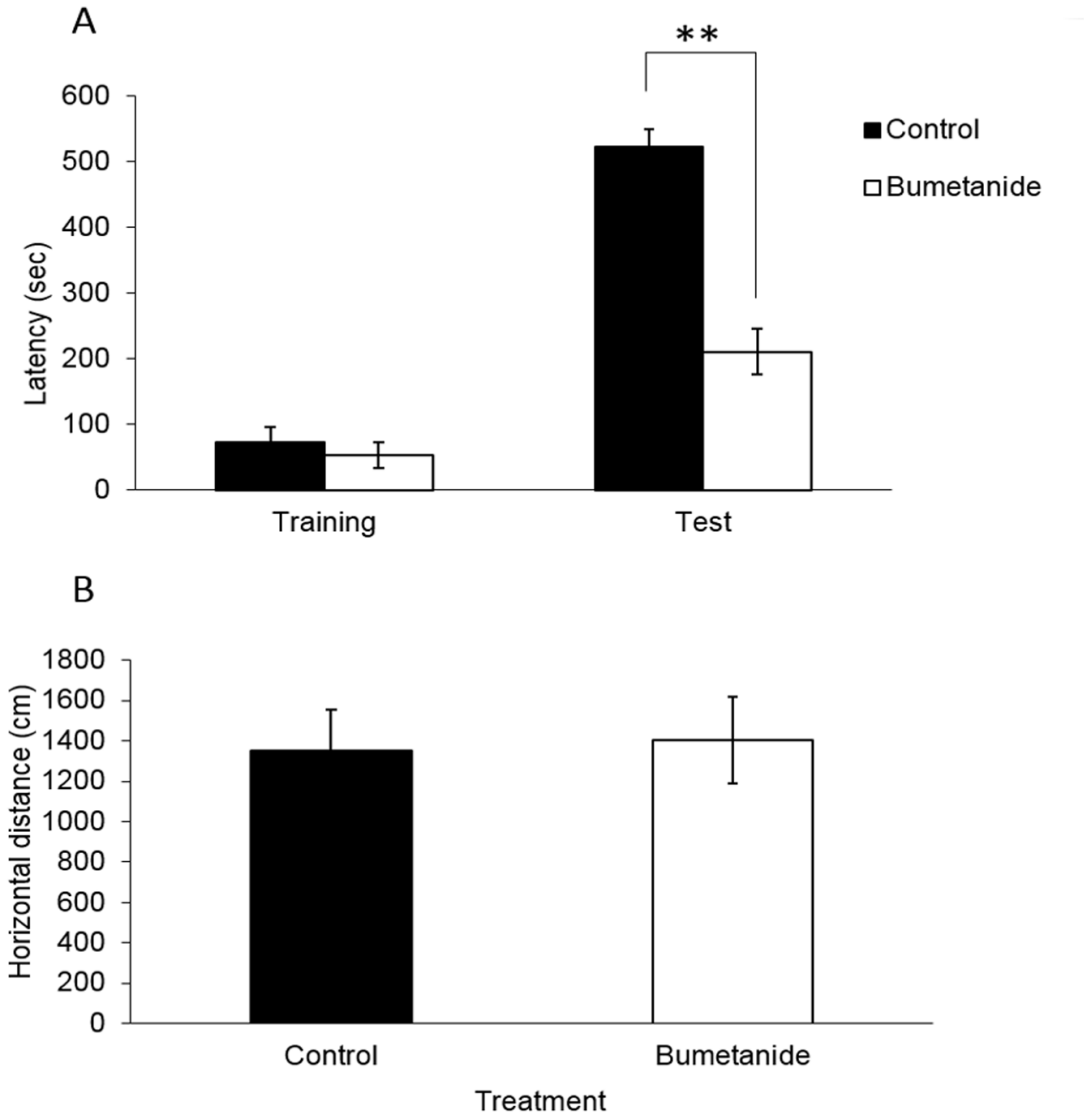
Systemic administration of bumetanide blocked inhibitory avoidance learning. (A) Rats were administered vehicle and bumetanide intravenously 1 hr prior to training session. The test was carried out 24 hrs later. Result showed that retention latency of the bumetanide-treated group was decreased significantly compared with vehicle-treated group (***p<0.001), n = 10 for each group. Data represent the medians ± interquartile range. (Mann-Whitney U-tests). (B) No significant difference had been found in locomotor activity among vehicle-treated and bumetanide-treated group. n = 8 for each group. Data represent the mean ± SEM.

Result of experiment-1 showed that suprafusion of bumetanide attenuated hippocampal LTP formation. We suggested that inhibitory avoidance task which is a hippocampus-dependent task (IA task) may also been altered. 20 animals were randomly assigned to two groups including vehicle-treated group and bumetanide treated group. Rats were trained on the IA task and received either intravenous injection of vehicle (n = 10) or bumetanide (n = 10) 30 min prior to the training. Memory retention was tested 24 hr later. Bumetanide-treated rats showed significantly lower retention latencies (mean  = 210.8±33.7 s) than vehicle-treated rats (mean  = 522.7±22.6 s; Figure 2A), suggesting that bumetanide-treated animals had an attenuated memory for the task (p<0.001). This result is consistent with the extracellular recording results and showing that bumetanide altered hippocampus synaptic function.

**Figure 3:**
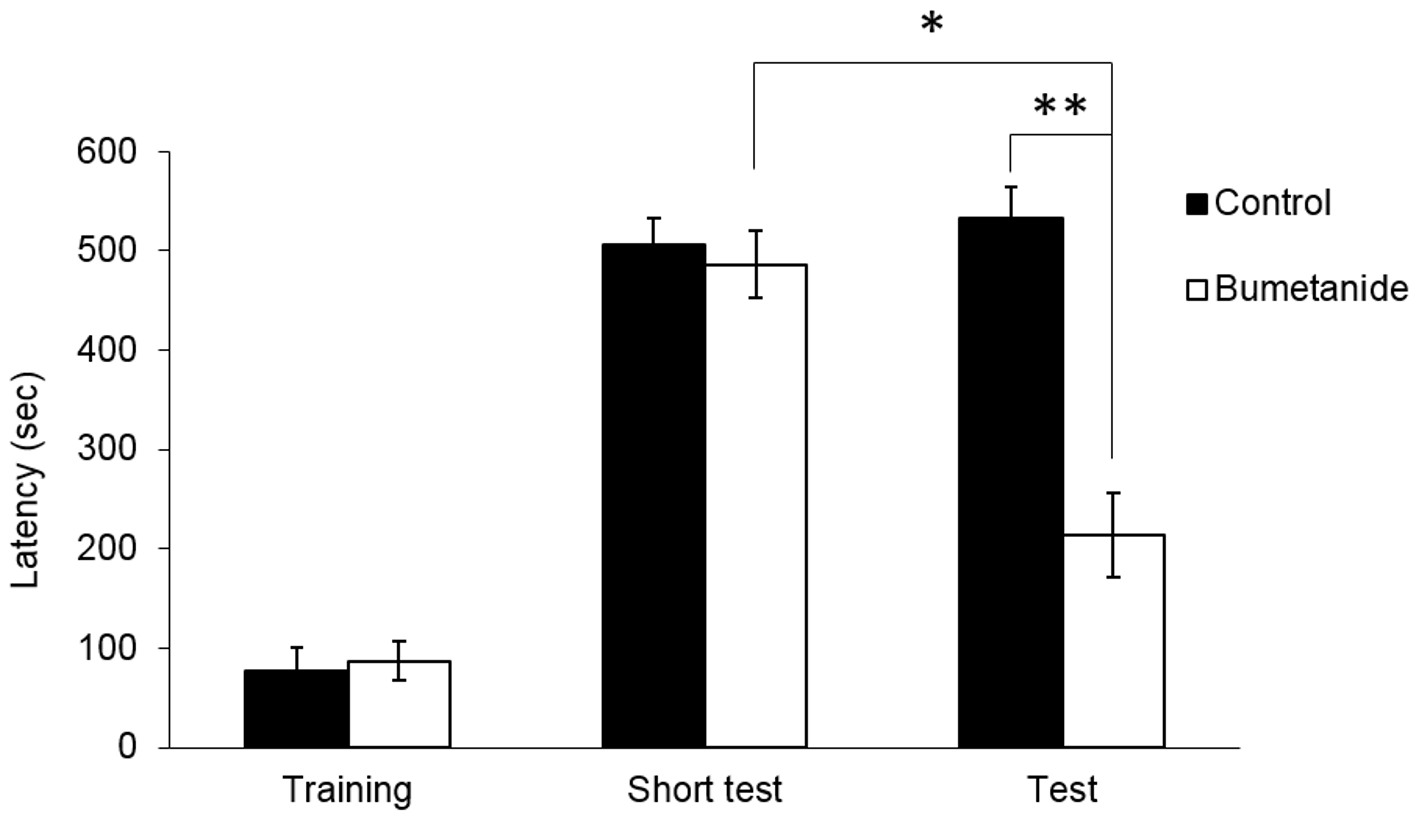
Systemic administration of bumetanide blocked the long-term memory but not the short-term memory formation. (A) Rats were administered vehicle and bumetanide intravenously 1 hour before training session. The 1^st^ and 2^nd^ test were carried out 1 hr and 24 hrs after training session respectively. Result showed that retention latency of the bumetanide-treated group was distinguishable with the vehicle-treated group. Significant difference had been found in the 2^nd^ test (**p<0.01) n = 8 for each group. Data represent the medians ± interquartile range (Mann-Whitney U-tests).

Bumetanide may have nonspecific effects on the locomotor activity during the test session, which may resulted in misinterpretation of its blockage effects on inhibitory avoidance learning. To exclude this possibility, we examined the animal's locomotor activity performance using the Student's t test. Our results of Student's t test for the total horizontal distance moved (Figure 3) did not reveal any significant difference between the two groups (p = 0.86).

### Experiment 3: Bumetanide blocked the long-term memory but not the short-term memory formation

This experiment was carried out to determine the blockage effect of bumetanide on inhibitory avoidance learning is on short-term memory or long-term memory formation. Sixteen animals were randomly distributed to two groups and received drug administration similar to experiment-1. An extra short-term memory test was taken place in 1 hr after training. Animals were then returned to their housing cage and subject for the 2^nd^ test 24 hrs after training. Bumetanide treated rats showed normal inhibitory avoidance learning in the short-term memory test. Rats given intravenous injection of bumetanide showed significantly lower retention latencies (mean  =  213.7 + 42.5 s) than rats given intravenous infusions of vehicle (mean =  533.1 + 31.3 s; Figure 3, p < 0.001) in the 2^nd^ test. Our results of Student's t test for the total horizontal distance moved (Figure 3B) did not reveal any significant difference between the two groups (p = 0.451). This result showed that the short-term memory remained intact in the bumetanide treated animals. The avoidance learning deficit may due to impairment on consolidation or retrieval of the long-term memory.

### Experiment-4: Systemic administration of bumetanide blocked avoidance learning induced MAPK phosphorylation in hippocampus

**Figure 4:**
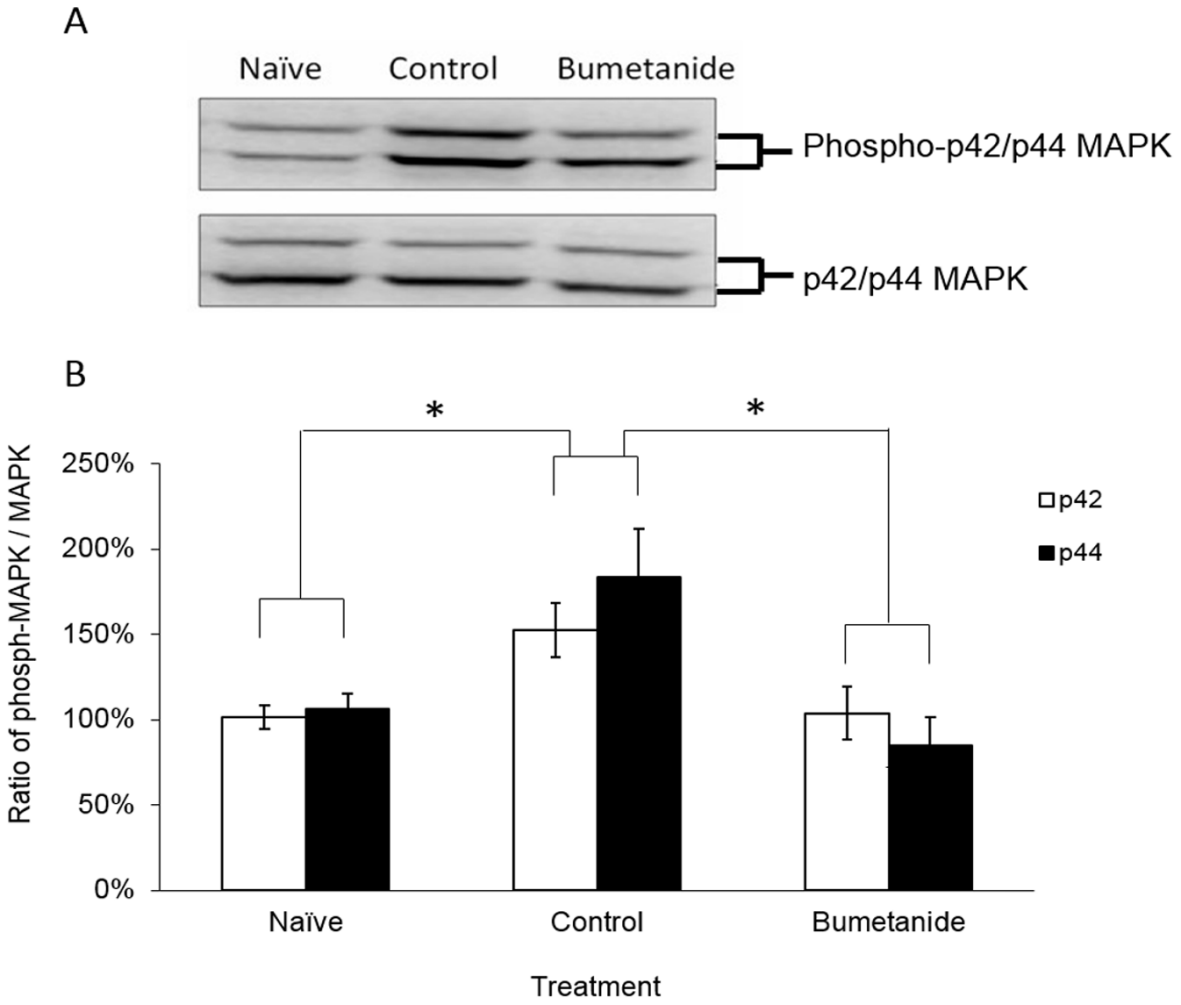
Systemic administration of bumetanide blocked avoidance learning induced MAPK activation in hippocampus. (A) Representative Western blots among different groups. (B) Densitometric analysis of the activation of MAPK in the hippocampus under different treatments. MAPK phosphorylation was significantly elevated in hippocampus after inhibitory avoidance learning. Bumetanide treatment significantly reduced the phosphorylation level of MAPK compared with vehicle-treated group (Values are mean ± SEM, * P<0.05 versus vehicle-treated group).

It is widely accepted that hippocampal MAPK signaling cascade is essential for the acquisition and consolidation of inhibitory avoidance memories. To study the possible involvement of MAPK signaling pathways on the attenuation effect of bumetanide in inhibitory avoidance learning, we used western blot to evaluate the hippocampal MAPK phosphorylation level. Eighteen animals were randomly assigned to three groups including naïve group, vehicle-treated group and bumetanide-treated group. The vehicle-treated and bumetanide-treated group received a single trial of inhibitory avoidance learning similar to experiment-1. Animals were sacrificed by decapitation 1 hour after the training session. The hippocampus was collected and subjected for western blot analysis. MAPK phosphorylation was significantly elevated in hippocampus after inhibitory avoidance learning (Figure 4A, lane 2) in the vehicle-treated group (p42: 153 ± 16%; p44: 184 ± 28%) compared with the naïve group (p42: 101 ± 7%; p44: 106 ± 9%). ANOVA analysis indicated a significant treatment effect [F(2, 15)  = 5.867, p = 0.042]. Bumetanide treatment significantly reduced the phosphorylation level of MAPK compared with vehicle-treated group (p42: 104 ± 16%; p44: 85 ± 16%) (p = 0.016). These results raise the possibility that the effect of bumetanide on avoidance learning is mediated by the hippocampal MAPK dependent signaling cascades.

### Experiment-5: Picrotoxin enhanced hippocampal fEPSPs were disrupted by suprafusion of bumetanide in a dose dependent manner

**Figure 5:**
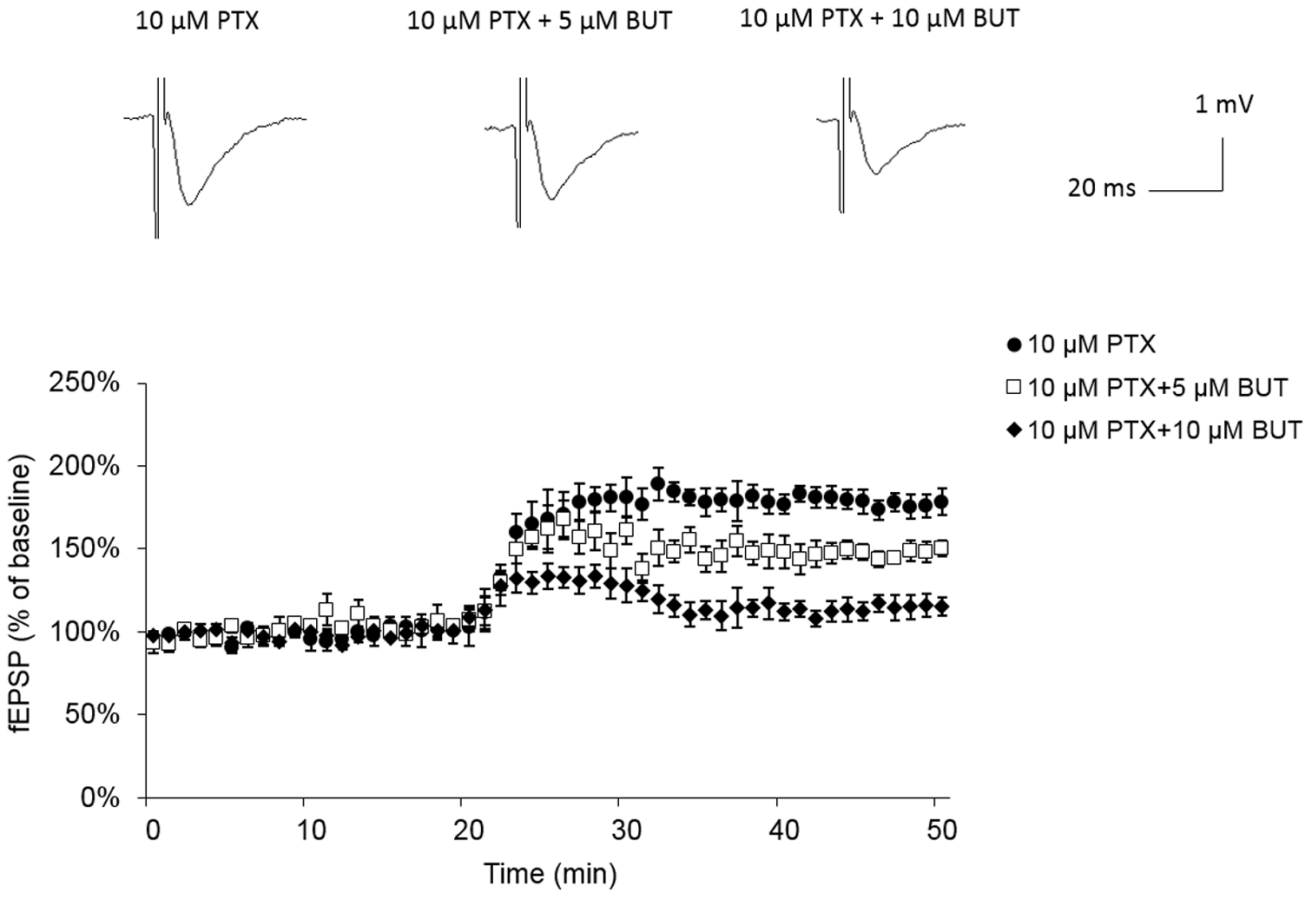
Picrotoxin enhanced hippocampal fEPSPs were disrupted by suprafusion of bumetanide in a dose dependent manner. (A) From Left to right, representative traces of fEPSPs recorded extracellularlly under control condition, after exposure to 10 µM picrotoxin (PTX), PTX + 5 µM bumetanide (BUT), and PTX + 10 µM BUT. Application of 10 µM PTX significant enhanced fEPSP amplitude in hippocampus CA1 Schaffer collateral fiber (178±8%, ***p<0.001 compared with control fEPSP). The picrotoxin enhanced fEPSPs were attenuated by bumetanide treatment in dose dependent manner (5 µM bumetanide: 149±10%, P = 0.066 compared with PTX group; 10 µM bumetanide: 117±9%, ***p<0.001 compared with PTX group, P = 0.453 compared with control group).

To determine whether the GABAergic signaling is responsible for the bumetanide suppression effect on hippocampus function Additional rats were subjected for *in vitro* extracellular recording. Briefly, they were randomly assigned to four different treatments including control (vehicle), picrotoxin treated alone (PTX), PTX +5 µM bumetanide, and PTX +10 µM bumetanide. A subthreshold dose (10 µM) of picrotoxin, A GABA_A_ channel blocker was used to enhance the amplitude of hippocampal fEPSP. According to the results, in the present of 10 µM of picrotoxin (PTX group), the amplitude of hippocampal fEPSPs significant elevated when compared with control group (178±8%, ***p<0.001) (Figure 5). In addition, the picrotoxin enhance effect was attenuated by co-treatment with bumetanide in a dose dependent manner. (5 µM bumetanide: 149±10%, P = 0.066 compared with PTX group; 10 µM bumetanide: 117±9%, ***p<0.001 compared with PTX group, P = 0.453 compared with control group) (Figure 5). These results imply that the blockage effect of bumetanide on hippocampal LTP formation is probably via enhancing GABA_A_ inhibition.

## Discussion

In this study, we used inhibitory avoidance task, extracellular recording and western blot to evaluate the role of NKCC1 on hippocampus function. We have demonstrated that formation of hippocampal LTP is significantly blocked in bumetanide-treated slices dose-dependently. This impairment of LTP may be occurs at the postsynaptic level since we did not detect significant differences in the synaptic volley and fEPSP ratio. Our results showed that intravenous administration of bumetanide 30 min prior to the learning phase blocked inhibitory avoidance learning. No significant differences had been observed in either retention latency in training phase or total distance of horizontal movement. These results indicated that the inhibition effect of bumetanide on avoidance learning was not the result of impaired locomotor activity. Subsequent experiment's results indicated that the inhibition effect of bumetanide on the avoidance learning was due to impairment on consolidation. Finally, western blot results revealed that hippocampal MAPK activation was attenuated after avoidance learning in bumetanide-treated animals.

It should be notified that, besides the potential for impaired locomotor activity, which we have control for, there are other possible explanations besides blocked learning that may need to be considered. For example, are the rats equally sensitive to shock? Is their visual system intact? Further experiments will be needed to determine whether bumetanide had non-specific effects which may alter the nociception and/or visual function of the animals.

Our first major concern regarding the interpretation of our results is that the acute bumetanide effects observed here might have been the result form some systemic effect rather that from a CNS-specific effect pharmacological action of the bumetanide on neuronal or glial NKCC1. We concluded that this possibility is an unlikely explanation for our results, since we used a low dose are lower by comparison to the clinical dose when used as diuretics for human patients. In our experiment no renal effect of bumetanide had been found (data not shown). We suggest that the possibility of systemic effect could be more definitively tested, for example, by using intracranial infusion, or by using other diuretics that have no affinity for the NKCC1.

Our second major concern is that it's known that the rat, unlike the human, rapidly transforms almost all of the bumetanide delivered in a given dose into a host of metabolites [22]. Is it possible that the effects of one of these rat-specific metabolites was responsible, rather than bumetanide itself, or is the result an artifact of something unrelated to bumetanide? In order to address this issue, it would be helpful to do additional experiments at more appropriate time intervals and doses. For example, doing the training session at an earlier time following bumetanide administration, such as at 20 minutes, and using a larger dose of bumetanide, which would more likely represent a time at which there would be significant levels of bumetanide in the plasma and brain, and at some later time such as 3 or 4 hours, to control for the issue that the major effect might be due to circulating metabolites rather than bumetanide itself.

NKCC1 is essential for ion homeostasis and volume regulation in neurons and astrocytes and is regulated by various neurotransmitters and hormones including glutamate and estradiol [23], [24]. Pervious results showed the NKCC1 activity is stimulated by glutamate through activation of N-methyl-D-aspartate, α-amino-3-hydroxy-5-methylisoxazole-4-propionate, and metabotropic glutamate receptors [25], [26]. However, NKCC1 also stimulates glutamate release [27]. During ischemia or TBI, the extracellular potassium concentration is elevated and induces glutamate release, which is mediated by volume-sensitive NKCC1. Blockade of NKCC1 activity by bumetanide significantly decreases the glutamate release [28], [29]. The interaction between NKCC1 and glutamate exacerbates the NKCC1 overexpression and brain edema formation during TBI. Our previous results had proven that systemic administration of bumetanide significantly attenuated traumatic brain injury induced neuronal damage and brain edema [17]. It is well known that hippocampal glutamergic synaptic transmission is essential for the formation of associative learning. It may explain the blockage effect of bumetanide on inhibitory avoidance learning. As we mentioned above, minor changes in the intracellular chloride concentration by blocking NKCC1 can significantly affect the strength, and even polarity, of GABAergic neurotransmission [2], [3]. Our results showed that input/output curves for extracellular fEPSP were indistinguishable between slices from vehicle-treated and bumetanide-treated group. The slopes of bumetanide-treated slice's fEPSP responses were not significantly different from the fEPSP responses of vehicle-treated slices. This result indicates that the given dose of bumetanide treatment what we used in this study did not modify the basal synaptic transmission at the postsynaptic level.

Our result is consistent with previous observation that hippocampal MAPK signal cascade is essential for the consolidation of associative learning [30]. The most important question emanating from this study relates to the mechanism by which the stimulation of NKCC1 controls the MAPK cascade phosphorylation. Our previous studies demonstrated that the NKCC1 blockage attenuated the neuronal damage and brain edema after traumatic brain injury by decreasing the phosphorylation of Raf/MEK/ERK cascade proteins. Taken together, the activation of NKCC1 might trigger the release of glutamate. In neurons, MAPKs can be activated by stimulation of glutamatergic NMDA receptors [30], [31] and results in calcium influx [32], [33]. Therefore, NKCC1 stimulation may increases intracellular free [Ca^2+^] via the activation of hippocampal glutamate NMDA receptors and is proposed to affect the signal transduction pathway either by a direct effect on one of its proteins or by Ca^2+^ activation of certain isoforms of the protein kinase family [34], [35], [36]. Further studies are necessary to elucidate the mechanism in detail. It should be mention that we only administered bumetanide intravenously. We cannot exclude the possibility that other brain regions are also involved in the bumetanide blockage effect on inhibitory learning. Further experiments such as local infusion of bumetanide into these regions are required to clarify the possible role of other brain regions in the bumetanide blockage effect on inhibitory learning.

Recently, Krystal and colleagues reported that loop diuretics had anxiolytic effects in rat models of conditioned anxiety [37]. In their study, two diuretic including bumetanide and furosemide were tested by using standard anxiety models. They found either furosemide or bumetanide significantly reduced conditioned anxiety in the contextual fear-conditioning and fear-potentiated startle models. These results implicate the cation-chloride cotransport system as possible molecular mechanism involved in anxiety, and as novel pharmacological target for the development of anxiolytics. The clinical potential of loop diuretics for treating some types of anxiety disorders deserves further investigation.

## Conclusions

In conclusion, we reported that in a lower dose of bumetanide had effects on the hippocampal function. Either inhibitory avoidance learning or hippocampal LTP formation had been impaired. Our findings match previous findings by indicating that hippocampal MAPK activity can be modulated by pharmacological interventions of NKCC1 inhibitor.
